# A Comparison of a Machine Learning Model with EuroSCORE II in Predicting Mortality after Elective Cardiac Surgery: A Decision Curve Analysis

**DOI:** 10.1371/journal.pone.0169772

**Published:** 2017-01-06

**Authors:** Jérôme Allyn, Nicolas Allou, Pascal Augustin, Ivan Philip, Olivier Martinet, Myriem Belghiti, Sophie Provenchere, Philippe Montravers, Cyril Ferdynus

**Affiliations:** 1 Réanimation Polyvalente, Centre Hospitalier Universitaire Félix Guyon, Saint-Denis, France; 2 Département d'Anesthésie Réanimation, APHP, CHU Bichat-Claude Bernard, Paris, France; 3 Département d'Anesthésie Réanimation, Institut Mutualiste Montsouris, 42 boulevard Jourdan, Paris, France; 4 Université Denis Diderot, PRESS Sorbonne Cité, Paris, France; 5 Unité de Soutien Méthodologique, CHU de La Réunion, Saint-Denis, France; 6 INSERM, CIC 1410, Saint-Pierre, France; University of Milano, ITALY

## Abstract

**Background:**

The benefits of cardiac surgery are sometimes difficult to predict and the decision to operate on a given individual is complex. Machine Learning and Decision Curve Analysis (DCA) are recent methods developed to create and evaluate prediction models.

**Methods and finding:**

We conducted a retrospective cohort study using a prospective collected database from December 2005 to December 2012, from a cardiac surgical center at University Hospital. The different models of prediction of mortality in-hospital after elective cardiac surgery, including EuroSCORE II, a logistic regression model and a machine learning model, were compared by ROC and DCA. Of the 6,520 patients having elective cardiac surgery with cardiopulmonary bypass, 6.3% died. Mean age was 63.4 years old (standard deviation 14.4), and mean EuroSCORE II was 3.7 (4.8) %. The area under ROC curve (IC95%) for the machine learning model (0.795 (0.755–0.834)) was significantly higher than EuroSCORE II or the logistic regression model (respectively, 0.737 (0.691–0.783) and 0.742 (0.698–0.785), p < 0.0001). Decision Curve Analysis showed that the machine learning model, in this monocentric study, has a greater benefit whatever the probability threshold.

**Conclusions:**

According to ROC and DCA, machine learning model is more accurate in predicting mortality after elective cardiac surgery than EuroSCORE II. These results confirm the use of machine learning methods in the field of medical prediction.

## Introduction

Cardiac surgery is at high risk of intraoperative and postoperative complications. The benefit of surgery is sometimes difficult to predict and the decision to proceed to surgery on an individual basis is complex.

European recommendations cite various risk stratification methods in decision making, even though these scores cannot replace clinical judgment and multidisciplinary dialogue [1]. Among the many scores that have been proposed, the European EuroSCORE II and the American Society of Thoracic Surgeons (STS) scores have been the most studied, and are the most widely used [2–5].

EuroSCORE II, the replacement for EuroSCORE I, was developed and validated in 2012 from a cohort of more than 22,000 patients hospitalized in 154 hospitals in 43 countries, and is used to predict postoperative mortality during hospital stay, through the collection of preoperative variables [2]. Logistic regression was used to create EuroSCORE II and Receiver Operating Characteristic (ROC) analysis was used to evaluate the model. Several studies have shown the limits of this score in some surgeries or patient subgroups [6–8].

Machine learning, a subfield of artificial intelligence, is a relatively new method issued from the development of complex algorithms and the analysis of large datasets. Machine learning can establish powerful predictive models, and is already used by major companies such as Google and Facebook. Several studies have shown the superiority of machine learning over traditional logistic regression used in EuroSCORE II [9–13].

Moreover, Decision Curve Analysis (DCA) has been developed to evaluate and compare diagnostic and prediction models by integrating the clinical consequences of false positives and false negatives [14]. In addition to its many other advantages, this method takes into consideration the patient’s choice to put themselves at a risk of false negatives or false positives. The analysis is presented as a graph with the selected probability threshold plotted on the abscissa and the benefit of the evaluated model on the ordinate. This evaluation method may be of considerable help in making decisions on medical treatment with serious side effects, and several studies have been conducted in cancer diagnosis [15–18].

Our objective was to compare a machine learning-based model with EuroSCORE II to predict mortality after elective cardiac surgery, using ROC and decision curve analysis. To the best of our knowledge, machine learning and decision curve analyses have never previously been used in such context.

## Methods

### Study Design and Data Collection

Data from consecutive patients who underwent cardiac surgery with cardiopulmonary bypass between December 2005 and December 2012 in a 1200-bed university hospital were included in this single-center cohort study. Perioperative characteristics were obtained from our local, prospectively collected database (S1 Table). The study was approved by the local ethics committee, which waived the need for informed consent, because of the observational nature of the study (Institutional Review Board 00006477, Paris 7 University, AP-HP). Reporting of this study complies with the Strengthening the Reporting of Observational studies in Epidemiology recommendations statement for reporting.

EuroSCORE I and EuroSCORE II scores were calculated using the coefficients described in the literature [2,19]. We used socio-demographic characteristics, comorbities, data on pre-operative conditions, and EuroSCORE I and EuroSCORE II as predictors.

Patients with non-elective cardiac surgery were excluded from the analysis.

Perioperative care (including anaesthesia, monitoring techniques and normothermic cardiopulmonary bypass) was standardized for all patients.

### Models Development

The database was split into 2 datasets: 70% for models training and 30% for models validation. Each model was trained using a 5-fold cross validation process. For each model, we repeated this process 10 times to obtain 10 individual probabilities. The final individual probability for each model was the mean of these 10 individual probabilities.

### Logistic Regressions

Three logistic regressions were performed: two univariate models and one multivariate model. The first univariate model was adjusted using EuroSCORE I. The second model was adjusted using EuroSCORE II. In contrast to the sample used to assess EuroSCORE II, in our sample there were no patients requiring emergency surgery. We therefore fitted a third logistic regression model (named LR model), adjusted using EuroSCORE II covariates [2]. All covariates were kept in the model.

### Gradient Boosting Machine (GBM)

Gradient boosting machine is a family of supervised machine learning techniques for regression and classification problems that are highly customizable. Gradient boosting machine produces a prediction which is an ensemble of weak prediction models (*e*.*g*. decision trees) [20]. Using boosting techniques and a meta-algorithm for reducing bias, GBM can convert a set of weak predictors to strong ones [20].

### Random Forests (RF)

Random Forests are an ensemble learning method for classification and regression analysis. The principle of RF is to construct multiple decision trees and return the mode of these classes (classification) or mean prediction (regression) of the individual trees. In contrast to classical decision trees, RF are more robust to overfitting [20].

### Support Vector Machine

Support vector machine is a class of supervised learning models for regression and classification analysis [20]. Support vector machine separates the input feature space in hyperplanes. Support vector machine can handle linear and nonlinear separation of the feature space using kernels [20]. Support vector machine offers good performance but can be computational complex with high dimensional input space features and nonlinear kernels.

### Naïve Bayes Model

The Naïve Bayes model is a supervised learning method for classification analysis based on Bayes' theorem [20]. Naïve Bayes assumes that given a class of the outcome, all covariates are independent (naïve assumption). With appropriate preprocessing, a simple Naïve Bayes classification can outperform more advanced machine learning methods [21].

### Features Selection

Some of the methods are sensitive to correlation between input features [20,21]. To overcome this problem we fitted the four models described above (GBM, RF, Support vector machine and Naïve Bayes) twice. For the first fit, we kept all input features in the analysis. For the second fit, we applied Chi-Square filtering to the input features and then used only relevant features in each of the four models.

### Ensemble of Models

It has been shown that assembling the results of different machine learning algorithms can outperform any single base learner [20]. In this way, we performed a greedy ensemble (*i*.*e*. a weighted sum) of the probabilities obtained with each of the four machine learning algorithms (GBM, RF, Support vector machine and Naïve Bayes), which we called ML model.

### Models Performance

We assessed the performance of each model using the area under the ROC curves (AUC) on the validation dataset. We used bootstrap with 1000 replications to obtain 95% Confidence Intervals (95% CI). Comparisons between ROC Curves were performed using the method described by DeLong *et al*. [22].

### Statistical Analysis

Qualitative variables were expressed as frequencies, percentages, and 95% confidence intervals (95% CI). Quantitative variables were expressed as mean and standard deviation (SD) or median and interquartile range (IQR). Comparisons of percentages were performed with a Chi-square test or by Fisher’s exact test, as appropriate. Comparisons of means were performed by Student t-test or by Mann and Whitney test, as appropriate.

Finally, we analyzed the net benefit of EuroSCORE II, LR model and ML model for predicting postoperative mortality. For these three methods, we calculated the net benefit using DCA, as described by Vickers *et al*.: it consists in the subtraction of the proportion of all patients who are false-positive from the proportion who are true-positive, weighting by the relative harm of a false-positive and a false-negative result [14].

$$Net\;benefit=\frac{True\;Positives}{n}-\left(\frac{Pt}{1-Pt}\right)\frac{False\;Positives}{n}$$

where n is the total number of patients included in the study and Pt is the probability threshold [14,23,24]. For example, a net benefit of 0.08 for a Pt of 50% can be interpreted as: « using this predictive model, 8 patients per 100 patients who will die after elective cardiac surgery will not have surgery without increasing number of not operated patient undue». The decision curve is constructed by varying Pt and plotting net benefit on the y vertical axis against Pt on the x horizontal axis.

DCA was performed using the SAS macro provided by Vickers *et al*. All statistical tests were performed at the two-tailed level of significance at 5%. All analyses were performed using SAS 9.4 (SAS Institute, Inc, Cary, NC, USA) and R software version 3.2.2 (The R Foundation for Statistical Computing; Vienna, Austria) with packages XGBoost, ExtraTrees and e1071 [25–27].

## Results

### Characteristics of Patients

From December 2005 to December 2012, 6,889 cardiac procedures with cardiopulmonary bypass were performed including 369 (5.3%) patients with non-elective cardiac surgery that were excluded from the analysis. The remaining 6,520 patients constituted the cohort.

Pre and intra-operative characteristics of the 6,520 patients are presented in Table 1. Among them, 411 (6.3%; 95%CI: 5.7–6.9) died during the in-hospital stay. The mean (standard deviation) age was 63.4 (14.4) years, and the mean EuroSCORE II was 3.7 (4.8) %. Univariate analysis showed a significant difference of mortality for 50 variables: age, height, weight, body mass index, 24 comorbities, 4 preoperative treatments, 8 preoperative paraclinic data, 5 preoperative coronarography data, and 5 surgery characteristics (Table 1).

**Table 1:** Characteristics of patients at admission and evolution in ICU (whole dataset).

	Missing data	Total (n = 6,520)	Alive (n = 6,109)	Dead (n = 411)	*P-value*
Age, mean (sd), years	0	63.4 (14.4)	63.1 (14.5)	68.2 (13.4)	< 0.0001
Sex (male), n (%)	0	4449 (68.2%)	4178 (68.4%)	271 (65.9%)	0.30
Weight, mean (sd), kg	14	75.4 (14.9)	75.6 (14.9)	72.4 (15.0)	< 0.0001
Height, mean (sd), cm	19	168.5 (9.5)	168.6 (9.5)	166.7 (9.8)	< 0.0001
BMI, mean (sd), kg.m^-2^	19	26.5 (4.6)	26.5 (4.6)	26.0 (4.9)	0.02
Comorbidities, n (%)					
Gastroduodenal ulcer	0	288 (4.4%)	265 (4.3%)	23 (5.6%)	0.23
CancerNoYes, > 5 years agoYes, < 5 years agoUncontrolled without metastasesUncontrolled with metastases	0	6002 (92.1%)238 (3.6%)195 (3.0%)66 (1.0%)19 (0.3%)	5654 (92.5%)210 (3.4%)169 (2.8%)60 (1.0%)16 (0.3%)	348 (84.7%)28 (6.8%)26 (6.3%)6 (1.5%)3 (0.7%)	< 0.0001
Liver cirrhosisNoYes, uncomplicatedYes, complicated	0	6455 (99.0%)40 (0.6%)25 (0.4%)	6056 (99.1%)32 (0.5%)21 (0.3%)	399 (97.1%)8 (1.9%)4 (1.0%)	0.0002
Immunodeficiency	0	88 (1.3%)	73 (1.2%)	15 (3.7%)	< 0.0001
Chronic pulmonary disease	0	375 (5.8%)	330 (5.4%)	45 (10.9%)	< 0.0001
Chronic obstructive pulmonary disease	0	642 (9.9%)	561 (9.2%)	81 (19.7%)	< 0.0001
Diabetes mellitus	2	1674 (25.7%)	1547 (25.3%)	127 (31.0%)	0.01
Diabetes mellitus with insulin	2	513 (7.9%)	459 (7.5%)	54 (13.2%)	< 0.0001
Hypertension	0	3678 (56.4%)	3427 (56.1%)	251 (61.1%)	0.05
Smoking (current or former)	25	3336 (51.4%)	3131 (51.4%)	205 (50.5%)	0.72
Peripheral vascular disease	0	905 (13.9%)	829 (13.6%)	76 (18.5%)	0.005
Chronic kidney disease requiring dialysis	0	57 (0.9%)	47 (0.8%)	10 (2.4%)	0.003
Dyslipidemia	0	3288 (50.4%)	3100 (50.7%)	188 (45.7%)	0.05
History of coronary artery disease	0	2496 (38.3%)	2351 (38.5%)	145 (35.3%)	0.19
Myocardial infarction < 90 days	0	440 (6.7%)	411 (6.7%)	29 (7.1%)	0.80
Previous cardiac surgery	0	627 (9.6%)	532 (8.7%)	95 (23.1%)	< 0.0001
History of endocarditis	0	192 (2.9%)	171 (2.8%)	21 (5.1%)	0.007
History of cardiac congestive failure	0	1047 (16.1%)	919 (15.0%)	128 (31.1%)	< 0.0001
Angor Canadian Cardiovascular Society class01234	0	4919 (75.4%)165 (2.5%)949 (14.6%)436 (6.7%)51 (0.8%)	4588 (75.1%)159 (2.6%)912 (14.9%)408 (6.7%)42 (0.7%)	331 (80.5%)6 (1.5%)37 (9.0%)28 (6.8%)9 (2.2%)	< 0.0001
History of thromboembolic event	0	353 (5.4%)	317 (5.2%)	36 (8.8%)	0.002
Hemorrhagic stroke	0	57 (0.9%)	52 (0.8%)	5 (1.2%)	0.44
Ischemic stroke	0	464 (7.1%)	415 (6.8%)	49 (11.9%)	< 0.0001
Poor mobility	0	179 (2.7%)	161 (2.6%)	18 (4.4%)	0.04
Mediastinal radiotherapy	0	122 (1.9%)	111 (1.8%)	11 (2.7%)	0.21
Supraventricular arrhythmiaNoYes, paroxysmalYes, permanent	0	5148 (79.0%)646 (9.9%)726 (11.1%)	4886 (80.0%)570 (9.3%)653 (10.7%)	262 (63.7%)76 (18.5%)73 (17.8%)	< 0.0001
Ventricular arrhythmia	0	103 (1.6%)	95 (1.6%)	8 (1.9%)	0.54
Pacemaker	0	222 (3.4%)	189 (3.1%)	33 (8.0%)	< 0.0001
Atrial fibrillation	0	776 (11.9%)	696 (11.4%)	80 (19.5%)	< 0.0001
New York Heart Association class1234	0	1374 (21.1%)603 (9.2%)214 (40.1%)1929 (29.6%)	1307 (21.4%)587 (9.6%)2496 (40.9%)1719 (28.1%)	67 (16.3%)16 (3.9%)118 (28.7%)210 (51.1%)	< 0.0001
Congestive heart failure	0	255 (3.9%)	200 (3.3%)	55 (13.4%)	< 0.0001
Active endocarditis	0	202 (3.1%)	172 (2.8%)	30 (7.3%)	< 0.0001
Critical preoperative state	0	107 (1.6%)	71 (1.2%)	36 (8.8%)	< 0.0001
Preoperative treatment, n (%)					
Antiplatelet therapyNoneAspirin onlyOthers	0	2716 (41.7%)2666 (40.9%)1138 (17.4%)	2526 (41.3%)2519 (41.2%)1064(17.4%)	190 (46.2%)147 (35.8%)74 (18.0%)	0.08
Beta blocker	0	3887 (59.6%)	3661 (59.9%)	226 (55.0%)	0.05
Anti-arrhythmic	0	880 (13.5%)	796 (13.0%)	84 (20.4%)	< 0.0001
Statin	0	3837 (58.8%)	3636 (59.5%)	201 (48.9%)	< 0.0001
Diuretic	0	2359 (36.2%)	2125 (34.8%)	234 (56.9%)	< 0.0001
Calcium Channel Blockers	0	1298 (19.9%)	1217 (19.9%)	81 (19.7%)	0.92
Angiotensin-converting enzyme inhibitor	0	3313 (50.8%)	3117 (51.0%)	196 (47.7%)	0.19
Nitrate treatment	0	85 (1.3%)	79 (1.3%)	6 (7.1%)	0.77
Preoperative data’s					
Hemoglobin concentration, mean (sd), g.dL^-1^	20	13.3 (1.8)	13.4 (1.7)	12.4 (2.1)	< 0.0001
Platelet numeration, mean (sd), x10^9^.L^-1^	59	232.9 (78.3)	233.3 (77.3)	227.3 (91.1)	0.01
Prothrombin activity, mean (sd), %	91	83.3 (12.6)	89.8 (12.1)	84.5 (17.1)	< 0.0001
Serum creatinine, mean (sd), μg.L^-1^	27	99.5 (62.6)	97.8 (59.8)	124.8 (91.8)	< 0.0001
Creatinine clearance (Cockcroft), mean (sd), mL.min^-1^	37	77.7 (32.7)	78.9 (32.5)	60.3 (29.5)	< 0.0001
Creatinine clearance (MDRD), mean (sd), mL.min^-1^	38	75.7 (25.3)	76.5 (25.0)	63.9 (27.7)	< 0.0001
Left ventricular ejection fraction, mean (sd), %	2	57.9 (12.2)	58.1 (12.0)	54.7 (13.7)	< 0.0001
Systolic pulmonary arterial pressure, mean (sd), mm Hg	0	31.9 (12.6)	31.6 (12.3)	36.7 (15.0)	< 0.0001
Preoperative coronary angiography, n (%)					
Coronary disease	0	3253 (49.9%)	3069 (50.2%)	184 (44.8%)	0.03
Number of vessel-disease01234	0	3211 (49.2%)487 (7.5%)624 (9.6%)2079 (31.9%)119 (1.8%)	3002 (49.1%)445 (7.3%)588 (9.6%)1959 (32.1%)115 (1.9%)	209 (50.8%)42 (10.2%)36 (8.8%)120 (29.2%)4 (1.0%)	0.10
Valve disease	0	3810 (58.4%)	3526 (57.7%)	284 (69.1%)	< 0.0001
Functional tricuspid insufficiency	0	693 (10.6%)	630 (10.3%)	63 (15.3%)	0.001
Ascending aortaNoAneurysmDissection	0	6026 (92.4%)435 (6.7%)59 (0.9%)	5652 (92.5%)410 (6.7%)47 (0.8%)	374 (91.0%)25 (6.1%)12 (2.9%)	< 0.0001
Congenital heart disease	0	88 (1.3%)	87 (1.4%)	1 (0.2%)	0.05
Surgery characteristics, n (%)					
Number of surgical procedureisolated CABGSingle no-CABG2 procedures3 procedures	0	2504 (38.4%)2176 (33.4%)1511 (23.2%)329 (5.0%)	2399 (39.3%)2038 (33.4%)1388 (22.7%)284 (4.6%)	105 (25.5%)138 (33.6%)123 (29.9%)45 (10.9%)	< 0.0001
Coronary surgery	0	3127 (52.0%)	2953 (48.3%)	174 (42.3%)	0.02
Valve surgery	0	3649 (56.0%)	3376 (55.3%)	273 (66.4%)	< 0.0001
Aortic valve surgery	0	2382 (36.5%)	2214 (36.4%)	168 (40.9%)	0.06
Mitral valve surgery	0	1517 (23.3%)	1387 (22.7%)	130 (31.6%)	< 0.0001
Tricuspid valve surgery	0	765 (11.7%)	691 (11.3%)	74 (18.0%)	< 0.0001
Thoracic aorta surgery	0	569 (0.7%)	523 (8.6%)	46 (8.1%)	0.07

BMI: Body Mass Index; CABG: *Coronary artery bypass grafting*; MDRD: Modification of the Diet in Renal Disease.

### Receiver Operating Characteristic Analysis

Table 2 summarizes the performance of the different predictive models tested in the validation dataset: EuroSCORE I and II, the LR model, different machine learning techniques, and the ML model. The ML model was the most accurate (AUC, 0.795 (0.755–0.834)). Of the different machine learning techniques, Random Forest had the best AUC irrespective of whether filtering was used. The AUC of the EuroSCORE II and the LR model was significantly lower than the ML model (both, p < 0.0001).

**Table 2:** Comparison of model performances on the validation dataset.

	AUC	95%CI
EuroSCORE I	0.719	0.674–0.763
EuroSCORE II	0.737	0.691–0.783
Logistic Regression Model (EuroSCORE II covariates)	0.742	0.698–0.785
**Machine Learning algorithms without features filtering**		
Gradient Boosting Machine	0.786	0.748–0.826
Random Forests	0.786	0.747–0.825
Naïve Bayes	0.734	0.689–0.779
Support Vector Machine	0.753	0.710–0.797
**Machine Learning algorithms with Chi-square filtering**		
Gradient Boosting Machine	0.784	0.743–0.824
Random Forests	0.788	0.748–0.827
Naïve Bayes	0.750	0.708–0.793
Support Vector Machine	0.736	0.689–0.784
Ensemble of ML Algorithms: ML model	0.795†	0.755–0.834

ML: Machine Learning.

†P value < 0.0001 compared with EuroSCORE II and with Logistic Regression Model.

**Fig 1:**
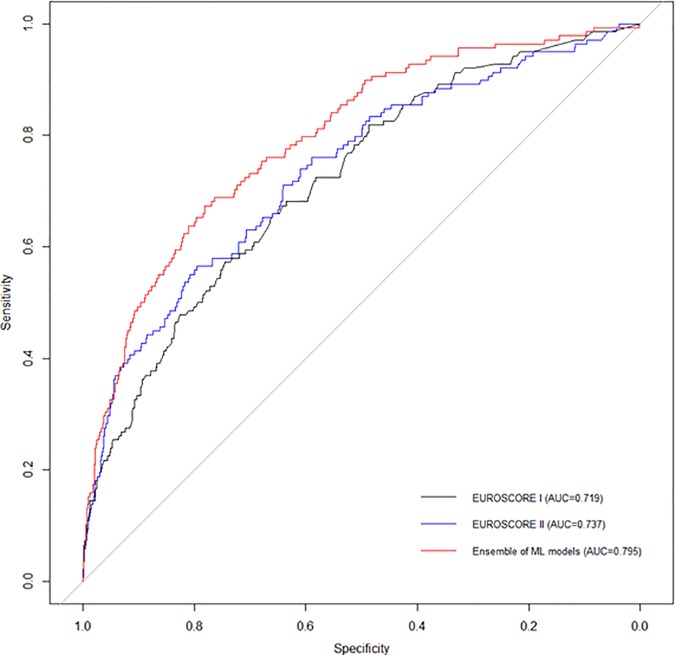
Receiver operating characteristic curves showing the performance of EuroSCORE I, EuroSCORE II, and the ML model in predicting post-operative mortality. Areas under curves (95% CI) are 0.719 (0.674–0.763), 0.737 (0.691–0.783), and 0.795 (0.755–0.834), respectively.

Fig 1 presents the ROC curves of EuroSCORE I and II, and ML model tested on the validation dataset, to predict mortality after cardiac surgery for all patients included in the study.

### Decision Curve Analysis

**Fig 2:**
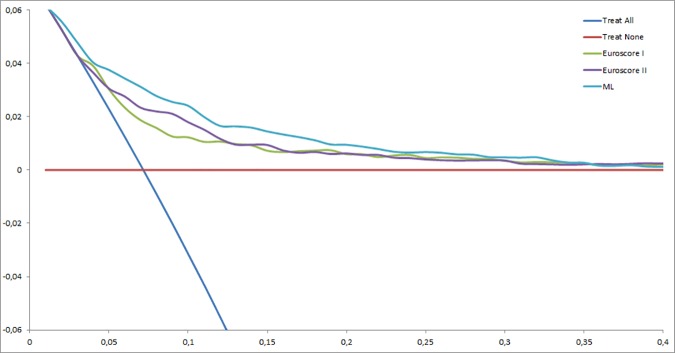
Decision curves showing the clinical usefulness of EuroSCORE I, EuroSCORE II, and the ML model in predicting post-operative mortality. The blue line represents the net benefit of providing surgery for all patients, assuming that all patients would survive. The red line represents the net benefit of surgery to none patients, assuming that all would die after surgery. The green, purple and turquoise lines represent the net benefit of applying surgery to patients according to EuroSCORE I, EuroSCORE II, and ML model, respectively. The selected probability threshold (*i*.*e*., the degree of certitude of postoperative mortality over which the patient's decision is not to operate) is plotted on the abscissa.

Fig 2 presents the decision curves that show the clinical usefulness of EuroSCORE I, EuroSCORE II and the ML model to predict mortality after cardiac surgery in the validation dataset. The results are presented as a graph with the selected probability threshold (*i*.*e*., the degree of certitude of postoperative mortality over which the patient's decision is not to operate) plotted on the abscissa and the benefits of the evaluated model on the ordinate [14,28]. The curves of EuroSCORE I and EuroSCORE II remain very close regardless of the threshold selected. The benefit of the ML model is always greater than that of EuroSCORE I and II regardless of the selected threshold, included between 1 and 6%.

## Discussion

To the best of our knowledge, this study is the first to use machine learning and DCA, relatively recent and efficient methods, in the context of cardiac surgery decision.

Mortality after this type of surgery is high. We report a mortality rate of 6.3%, which is slightly greater than reported in the EuroSCORE II study (4.6%), while we excluded urgent surgery. This difference may be related to a greater frequency of valvular surgery (56% and 46.3%) and less coronary isolated surgery (38.4% and 46.7%) in our cohort [2].

This study found the ML model to be superior to traditional logistic regression, which has already been observed in many studies [9–13]. For example, in the study of Churpek and *al*., machine learning and logistic regression were compared to predict clinical deterioration in a large multicentric database of 269,999 patients. Random Forest (AUC 0.80) was more accurate than logistic regression (AUC 0.77) in predicting clinical deterioration [9]. In the same way, the study de Taylor *et al*. on the prediction of mortality of 4676 patients with sepsis at the emergency department, found a superiority of their machine learning model (AUC 0.86) compared to their logistic regression model (AUC 0.76, p ≤ 0.003) [12].

EuroSCORE I was used during the years of patient inclusion in our database. Moreover, EuroSCORE I and EuroSCORE II were created from another database. It is why we created a logistic regression model from our database in order to not disadvantage EuroSCORE I and EuroSCORE II. Again, the ML model outperformed the LR model, with ROC analysis and DCA significantly more favorable for the ML model.

Beyond our study, it appears that machine learning may replace logistic regression for prediction when the dataset is several thousand patients or more, and when it is not necessary to assign a weight to each variable related criterion. In these cases, logistic regression may be always helpful.

The second aim of our study was to provide a complementary analysis of traditional ROC curves. This DCA provides complementary information which help in the decision making process involving exposure to risk in surgery. This technique is increasingly widely used and has already shown promise in cancer research. Our analyses show a moderate benefit from the different models (between 1 and 6%), even for the ML model. While the benefit is modest, the impact is major (*i*.*e*. prediction of mortality after surgery), and ML model is never inferior to EuroSCORE II. This point is interesting because European guidelines advocate the use of a prediction score to help in decision making [1].

Beyond the theory, combining these two innovative calculation and evaluation methods for predictive models could improve the clinical management of patients. Many predictive models, however, are of no use in clinical practice and we are waiting studies about utility of prediction scores in the area of preoperative cardiac evaluation.

Our study has several limitations. Firstly, because of the retrospective calculation of EuroSCORE II, bias may be present. Secondly, we were unable to gauge the degree of emergency as proposed in EuroSCORE II. This is a minor limitation because our objective was to help in decision making, and we acknowledge that decision making is different in an emergency situation. Third, this was a monocentric study, which could limit extrapolation of our ML model in another cardiac surgery center. Teams with sufficient data could establish their own ML model, or teams with multicentric data could conduct an even larger study than ours. Similarly, it would be interesting to use these methods in a more complex context, where standard scores are less efficient. For example, it may be very useful in decision making involving very old patients or to help choosing between transcatheter and surgical aortic valve replacement [6–8]. Finally, we cannot compare the ML model with other scores like the STS score, widely use in North America.

In conclusion, machine learning is more accurate than EuroSCORE II to predict mortality after non-urgent surgery. These results confirm the use of machine learning methods in the field of medical prediction. Decision curve analysis showed this prediction model to be a moderate help in decision making, according recommendations.

## Supporting Information

S1 Table Dataset. Patient’s data.
(XLSX)
